# Carriage rates and risk factors during an outbreak of invasive meningococcal disease due to *Neisseria meningitidis* serogroup C ST-11 (cc11) in Tuscany, Italy: a cross-sectional study

**DOI:** 10.1186/s12879-018-3598-3

**Published:** 2019-01-08

**Authors:** Alessandro Miglietta, Francesco Innocenti, Patrizio Pezzotti, Eleonora Riccobono, Maria Moriondo, Patrizia Pecile, Francesco Nieddu, Gian Maria Rossolini, Chiara Azzari, Meningococcal Carriage Study Group, Alessandra Bagnoli, Giuseppe Boncompagni, Francesco Cirpiani, Paolo Filidei, Giorgio Garofalo, Marinella Chiti, Gabriele Mazzoni, Astrid Mercone, Giovanna Mereu, Sabrina Novelli, Maria Grazia Santini, Maurizio Spagnesi, Emanuela Balocchini, Giovanni Rezza, Fabio Voller, Paola Stefanelli

**Affiliations:** 1 Department of Infectious Diseases, Istituto Superiore di Sanità, Viale Regina Elena, 299, 00161 Rome, Italy; 2 Regional Health Agency of Tuscany, Epidemiologic Observatory, Via Pietro Dazzi, 1, Flroence, Italy; 3 Units of Epidemiology and Preventive Medicine, Central Tuscany Health Authority, Via di San Salvi , 12 - Palazzina 16 –, 50135 Florence, Italy; 4 Clinical Microbiology and Virology Unit, Careggi University Hospital, Largo Brambilla, 3, 50134 Florence, Italy; 5 Laboratory of Immunology and Infectious Diseases, Anna Meyer Children’s University Hospital, University of Florence, Viale Gaetano Pieraccini, 24, 50139 Florence, Italy; 6 Department of Experimental and Clinical Medicine, University of Florence, Largo Brambilla, 3, 50134 Florence, Italy; 7 Living Environment, Food and Veterinary Prevention and Safety Office, Tuscany Region, Via Taddeo Alderotti, 26/N, 50139 Florence, Italy

**Keywords:** *Neisseria meninigitidis*, Carrier state, Cross-sectional studies, Disease outbreaks

## Abstract

**Background:**

During 2015–2016 an outbreak of invasive meningococcal disease due to *N. meningitidis* serogroup C ST-11 (cc11) occurred in Tuscany, Italy. The outbreak affected mainly the age group 20–30 years, men who have sex with men, and the area located between the cities of Firenze, Prato and Empoli, with discos and gay-venues associated-clusters. A cross-sectional-survey was conducted to assess the prevalence and risk factors for meningococcal-carriage, in order to address public health interventions.

**Methods:**

A convenience sample of people aged 11–45 years provided oropharyngeal swab specimens and completed questionnaires on risk factors for meningococcal carriage during a 3 months study-period, conducted either in the outbreak-area and in a control-area not affected by the outbreak (cities of Grosseto and Siena). Isolates were tested by culture plus polymerase chain reaction. Serogroup C meningococcal isolates were further characterized using multilocus sequence typing. Univariate and multivariate analyses were performed to estimate adjusted odds ratios (AORs) for meningococcal carriage.

**Results:**

A total of 2285 oropharyngeal samples were collected. Overall, meningococcal carriage prevalence was 4.8% (*n* = 110), with nonencapsulated meningococci most prevalent (2.3%; *n* = 52). Among encapsulated meningococci, serogroup B was the most prevalent (1.8%; *n* = 41), followed by serogroup Y (0.5%; *n* = 11) and serogroup C (0.2%; *n* = 4); one carrier of serogroup E and one of serogroup Z, were also found (0.04%). Three individuals from the city of Empoli were found to carry the outbreak strain, C:ST-11 (cc11); this city also had the highest serogroup C carriage prevalence (0.5%). At the multivariate analyses, risk factors for meningococcal carriage were: illicit-drugs consumption (AOR 6.30; *p* < 0.01), active smoking (AOR 2.78; *p* = 0.01), disco/clubs/parties attendance (AOR 2.06; *p* = 0.04), being aged 20–30 years (AOR 3.08; *p* < 0.01), and have had same-sex intercourses (AOR 6.69; *p* < 0.01).

**Conclusions:**

A low prevalence of meningococcal serogroup C carriage in an area affected by an outbreak due to the hypervirulent *N. meningitidis* serogroup C ST-11 (cc11) strain was found. The city of Empoli had the highest attack-rate during the outbreak and also the highest meningococcal serogroup C carriage-prevalence due to the outbreak-strain. Multivariate analyses underlined a convergence of risk factors, which partially confirmed those observed among meningococcal outbreak-cases, and that should be considered in targeted immunization campaigns.

**Electronic supplementary material:**

The online version of this article (10.1186/s12879-018-3598-3) contains supplementary material, which is available to authorized users.

## Background

*Neisseria meningitidis* is an obligate human commensal bacteria that colonizes the nasopharynx [1]. Colonization is a prerequisite for transmission and for developing invasive meningococcal disease (IMD). However, IMD is a rare consequence of invasion and in most individuals the colonization leads to a period of asymptomatic carriage after which *N. meningitidis* is naturally cleared, determining a serogroup-specific antibody response that acts as transitory immunization event [2].

Asymptomatic meningococcal carriage is recognized as an age-dependent phenomenon, with prevalence increasing through childhood from 4.5% in infants to a peak of 23.7% in 19-year olds and subsequently decreasing in adulthood to 7.8% in 50-year olds [3]. However, in closed/semi-closed settings and during outbreak-situations, the carriage rate may be higher [2].

The duration of the carriage state varies; it may be chronic, lasting for several months, intermittent or transient, and depends on the properties of the colonizing-strain [2]. One strain, the sequence-type 11 (ST11) clonal-complex (cc11) meningococci bearing serogroups C or W polysaccharide capsules, is recognized to have a high transmission and recovery rate to balance its short duration of carriage and inability to create a commensal relation with the host [4].

In Italy, the incidence rate (IR) of IMD is among the lowest in Europe [5, 6]. Until 2014, serogroup B was the main cause of IMD in the country; from 2015, serogroup C began predominating, determining the 44 and 43% of all IMD-cases in Italy in 2015 and 2016, respectively.

This sharp increase was driven by the Tuscany region that contributed for 48.4% (31/64) and 37.5% (30/80) of all IMD serogroup C cases (MenC) at national level in 2015 and 2016, respectively [6, 7]. The increased incidence of MenC reported by Tuscany was due to a clonal expansion of the hyper-virulent *N. meningitidis* strain C:P1.5–1,10–8:F3–6:ST-11(cc11) [8], which mainly affected the age group 20–30 years, men who have sex with men, and the area located between the cities of Firenze, Prato and Empoli, with discos and gay-venues associated-clusters [9]. As a consequence, in May 2015, a reactive immunization campaign offering a single dose of meningococcal serogroup C conjugate vaccine (MCC) or quadrivalent meningococcal conjugate vaccine (ACWY) to people aged 11–45 years, was implemented in Tuscany with the support of the Italian Ministry of Health (MoH). In addition, a targeted immunization programme for MSM was performed in late 2016.

Moreover, with the support of Italian National Institute of Health (Istituto Superiore di Sanità - ISS), the Tuscany Region conducted an outbreak investigation [9], and a cross-sectional carriage survey, whose results are presented in this paper. The primary objectives of the survey were to estimate the meningococcal carriage prevalence during the outbreak and to identify related risk factors, in order to address public health interventions.

## Methods

### Setting and outbreak-context

Tuscany is a region located in central Italy with about 3.8 million of inhabitants at the beginning of 2016, and the capital city is Florence [10].

MCC was introduced in Tuscany in 2006 with a single dose at 13–15 months of age, and a catch-up program was implemented in 2007 among those aged 11–20 years using ACWY. In addition, the Region introduced in 2017 a booster dose of MCC at 6–9 years of age.

The outbreak-response immunization programme was implemented in the whole Region through mass-media communications. People eligible for MCC/ACWY (aged 11–45 years) adhered to the campaign by going directly to immunization clinics.

As of 31st December 2017, vaccine coverage was 90.6% at 24 months of age, 53.6% in the age group 11–20 years, and 36.6% of the reactive vaccination campaign.

### Study design, sample size and sampling

This is a cross-sectional carriage survey conducted during the period March 1, 2016–June 30, 2016. The planned sample size of the study was of 2400 participants. Assuming a carriage prevalence of 18% [11, 12], this sample size had a precision of about 1.5% [i.e., the 95% Confidence Interval (95% CI) would have a range of about 3%]; in the worst case (i.e., carriage prevalence of 50%) the precision would be of about 2%. Recruiting occurred in four immunization clinics in the context of the immunization response campaign: two clinics, respectively located in the cities of Florence and Empoli (outbreak-area), and two in the cities of Grosseto and Siena (control-area). Individuals aged 11–45 years who presented at immunization clinics to adhere to the response campaign, were asked to participate in the study by the health personnel (Public Health Nurses).

### Specimen and data collection

A written informed consent form was signed by each participant, and consent was also obtained from parents/guardians of subject aged less than 18-years. After sample collection (done before vaccination), the recruited filled-in a self-administered questionnaire collecting demographic information and data on known risk factors for meningococcal carriage (e.g. alcohol-consumption and drinks-sharing, active and passive smoking, illicit-drugs use, disco attendance etc.) [13–17].

Oropharyngeal swab samples through the mouth were taken by Public Health Nurses of the vaccination clinic from the posterior wall of the oropharynx [18] and were immediately placed in a Copan Liquid Stuart’s swab [19] and transported within 4 h to the Clinical Microbiology and Virology Unit of Careggi University Hospital of Florence for culture, and immediately afterwards, to the Laboratory of Immunology and Infectious Diseases at the Anna Meyer Children’s University Hospital of Florence for polymerase chain reaction (PCR).

### Microbiological analyses

Samples were plated on Thayer-Martin agar and Chocolate agar (bioMeriéux, Marcy l’Étoile, France) using a Wasp processorTM (Copan Diagnostics Inc., Brescia, Italy) and incubated at 35 °C in a 5–10% CO2 atmosphere, as previously described by Gasparini et al. [20]. Morphological evaluation of bacterial colonies was performed after 24 and 48 h. All colonies suspected for being *N. meningitidis* were identified by MALDI-TOF (VITEK MS, bioMeriéux). Once confirmed as meningococci, colonies were subcultured on Chocolate agar to obtain pure cultures and stored at − 80 °C pending further investigations. Serogroups were assessed for each *N. meningitidis* isolate following the protocol proposed by the Center for Disease Control and Prevention (https://www.cdc.gov/meningitis/lab-manual/chpt10-pcr.html).

DNA from each sample was extracted and purified using the MagCore Genomic DNA Tissue Kit with automated Nucleic Acid Extractor HF16 (RBCBioscience, Taiwan). All samples were tested with real time-PCR (rt-PCR) for the *ctrA* gene [21]. Samples positive for *ctrA* gene were serotyped using rt-PCR (A, B, C, W, Y) and End-Point PCR (29E and Z) [22].

The double test (culture and PCR) was performed to maximize the sensitivity [23]. To assess the circulation of the outbreak strain, C:ST-11 (cc11), serogroup C meningococcal isolates were further characterized using multilocus sequence typing (MLST) according to the methods of Maiden et al. [24]. Alleles, sequence types and clonal complexes were assigned using the Neisseria species MLST database (http://pubmlst.org/neisseria).

### Data analysis

Questionnaires and results on carriage status communicated by laboratories were collected by the Regional Health Agency of Tuscany, entered into an Excel database through an optical reader and double checked by a statistician and an epidemiologist. After validation, the database was imported into STATA 12 software for data analysis.

Meningococcal carriage prevalence (overall and by serogroup) was computed and the exact binomial distribution was used to calculate 95% confidence intervals (CI).

The association between the study variables and meningococcal carriage state was assessed through univariate (Chi-Square Test) and multiple logistic models. For the latter, we firstly fitted multiple logistic regression models including all variables with a *p*-value < 0.20 at the univariate analysis. Secondly, backward selection was performed and only variables with a p-value < 0.10 (by the log-likelihood ratio test) were retained [25]. Age and sex were included in the final models independently from the estimated *p*-values at univariate analyses and in the backward selection. Because some categories had empty cells (i.e. no meningococcal carriage), in these cases we performed exact logistic regressions to assess the correlation.

Since only 4 serogroup C carriers were found, we used as outcome variable the carriage status (positive/negative, 1/0) regardless of serogroup, while the following were used as independent variables: gender; age-group (11–19; 20–30; 31–45), place (Siena, Grosseto, Firenze, Empoli) and month (March, April, May, June) of swab collection; occupation (grouped in those recognized in literature at higher risk for meningococcal carriage [13–17]: students, workers in restaurant/bar/pub/café, teachers, armed forces, health professionals, and other occupation); quantity and type of alcoholics consumed in a week (grouped in low, moderate and high drinking-risk-level by using the European Commission classification for alcohol-consumption [26]); active and passive smoking; and whether in the month before swab collection the recruited: shared drinks, consumed illicit-drugs, had sexual intercourses (including intimate kissing partners) with men and/or women (then grouped in no-sexual intercourses; heterosexual-intercourses; same-sex intercourses), used antibiotics, had upper respiratory tract infections and attended (a) discos/clubs/parties (b) bar/pub/restaurants (c) other close-groups (e.g. sporting groups).

Results were expressed in terms of odds ratios (ORs) and adjusted odd ratios (AORs), with 95% CI. Statistical significance was set at *p*-value< 0.05.

## Results

A total of 2285 oropharyngeal samples were collected (95.2% of the planned sample size).

The overall prevalence of *N. meningitidis* carriage was 4.8% (*n* = 110; 95% CI 2.0–5.3%), and the majority carried nonencapsulated meningococci (2.3%; 95% CI 1.7–3.1%). Among encapsulated meningococci, serogroup B was the most prevalent (1.8%; 95% CI 1.3–2.4%; *n* = 41), followed by serogroup Y (0.5%; 95% CI 0.2–0.9%; n = 11) and serogroup C (0.2%; 95% CI 0.0–0.4; n = 4). One carrier of serogroup E and one of serogroup Z were also found (0.04%; 95% CI 0.0–0.2%).

At the MLST analysis performed on the 4 *N. meningitidis* serogroup C isolates, three individuals from the city of Empoli (outbreak-area) were found to carry the outbreak strain, C:ST-11 (cc11), and one form the city of Grosseto (control-area) carried a C:ST-41/44 clonal complex meningococci.

Additional file 1: Table S1 describes the study-population showing the meningococcal carriage prevalence by serogroups, demographic characteristics and risk factors.

Each study centre contributed equally to the sample size, with serogroup C most prevalent in Empoli (0.5%). The 4 serogroup C carriers were all aged 11–19 years, students, and attended disco/clubs/parties in the month before swab collection.

Participants who shared drinks in the month before swab collection had a higher serogroups B and C carriage prevalence (2.7 and 0.4%, respectively) compared with participants who did not (1.2 and 0.0%, respectively).

Higher carriage prevalence was observed for all serogroups among illicit-drugs users, with serogroup Y most prevalent (11.4%).

The majority of smokers carried nonencapsulated meningococci (7.2%), while the 4 serogroup C carriers were all exposed to second-hand smoke.

Those who in the month before swab collection had same-sex intercourses showed a higher meningococcal carriage prevalence than those who had heterosexual or had not sexual intercourses, with nonencapsulated meningococci (76.2%) and *N. meningitidis* serogroups B and Y most prevalent (76.2, 4.8 and 4.8%, respectively).

Additional file 2: Table S2 shows the results of the univariate and multivariate analyses of factors associated with meningococcal carriage status.

At the multivariate analysis including variables with *p* < 0.20 at the unviariate analysis, factors statistically significant associated with the risk of being meningococcal carriage were: illicit-drugs consumption (AOR 6.30; 95% CI 2.44–16.32; *p* < 0.01), active smoking (AOR 2.78; 95% CI 1.37–5.63; *p* = 0.01), disco/clubs/parties attendance (AOR 2.06; 95%CI 1.04–4.23; *p* = 0.04), the age-group (p < 0.01) but with a significant association only for those aged 20–30 years (AOR 3.08; 95% CI 1.97–9.79), as well as having had sexual intercourse (p < 0.01) but with a significant increased risk only for those who had same-sex intercourses (AOR 6.69; 95% CI 2.16–20.70).

Similar results were observed at the multivariate analysis with backward elimination at 10% level, where the same variables remained correlated with meningococcal carriage status in the same direction of association.

## Discussion

This study shows meningococcal carriage prevalence and related risk factors during an outbreak due to the hypervirulent *N. meningitidis* serogroup C ST-11 (cc11) strain occurred in Tuscany, Italy, during 2015–2016 [8, 9]. Despite the outbreak, a low prevalence (0.2%) of serogroup C carriers was found. This finding is consistent with those of previous carriage surveys conducted during MenC-outbreaks, where serogroup C carriage prevalence found not to exceed 1%, being 0.3% in Canada [27], 1.0% in The Netherlands [28], 0.6% in Spain [29], 1.0% in Denmark [30], and 0.2% in Brazil [31]. This low serogroup C carriage prevalence during MenC-outbreaks, could be partially explained both by the hypothesis of Trotter et al. [32] that serogroup C strain have a high recovery and transmission rate, moving quickly through populations while maintaining a low prevalence, and by the hypothesis of Caugant et al. [4] that MenC-outbreaks, in particular due to hypervirulent strains (as ST11/cc11), follow their rapid spread in a specific-population in terms of asymptomatic carriers of short duration, as suggested by fact that these outbreaks are characterized by the occurrence of MenC-cases in different locations of restricted geographical areas over short periods of time, without apparent epidemiological links.

Both hypotheses imply that during MenC-outbreaks, there are population-pockets where transmission occurs with people becoming serogroup C carrier for a short period of time. These population-pockets, are difficult to detect through prevalence-surveys because this study-design provides a limited snapshot of a population, that also due to biases, in particular selection-bias, may not be reliable of the real carriage prevalence [1].

This limit is present in our study, because the convenience sample with recruitment at vaccination clinics, may have introduced selection biases by recruiting participants who were not in the affected population-pockets.

Moreover, the reactive vaccination campaign with MMC/ACWY, started 1-year before the study may have reduced the serogroup C meningococcal carriage prevalence, since meningococcal serogroup C conjugate vaccines have demonstrated a positive impact on reducing the carrier rate [33]. Despite this, it should be of note, that before, during and after this study, the MenC-outbreak in Tuscany continued to occur [9], suggesting that the bacterium was still circulating within specific population-pockets not surveyed.

On the other hand, a higher carriage prevalence due to nonencapsulated and serogroups B, Y meningococci was found, as in the aforementioned studies conducted during MenC-outbreak situations [27–31], indicating a more commensal behaviour and a longer duration of the carriage status, that facilitates the identification of people carrying these strains during carriage surveys [4, 32].

Differences in the meningococcal prevalence by serogroups, demographic characteristics and risk factors of the study population were observed.

In particular, according to the 2015–2016 MenC outbreak-investigation conducted by Miglietta et al. in Tuscany [9], where clusters were recognized among teenagers attending discos, the 4 serogroup C carriers were all aged 11–19 years and attended discos/club/parties during the month before swab collection. On the other hand, in contrast with the result of the outbreak-investigation, where an involvement of the MSM-community was also identified, none of the serogroup C carriers reported MSM-behaviour. This mismatch may be due to the low number of serogroup C carriers found that did not allow to clearly identify risk factors for serogroup C carriers during the outbreak, also determining the need to group all the serotypes together for the risk factors analyses.

Higher serogroup C carriage prevalence was observed in the city of Empoli; in line with this finding, this city was the one with the higher attack-rate during the 2015–2016 MenC-outbreak [9, 34]. In addition, the fact that the 3 carriers of the outbreak-strain (C:ST-11/cc11) were all from the city of Empoli and the one carrying a C:ST-41/44 clonal complex meningococci was found in Grosseto (control-area), strongly confirm the circulation of the outbreak-strain in the affected area.

Overall, risk behaviours identified through multivariate analyses confirmed what reported in the literature from carriage surveys. The age group 20-to-30 years was at increased risk for meningococcal carriage during outbreaks occurred in The Netherlands, Turkey, Wales, and in a Review of carriage studies conducted in Europe, and this is likely due to the convergence of risk behaviours among young adults [2, 15, 27, 28].

IMD-outbreaks were reported among illicit-drugs users in the United States (US) [35, 36] and this was a risk behaviour for meningococcal carriage reported in other surveys [14, 17]. The mechanisms through which illicit-drug use may contribute to carriage, meningococcal transmission, or development of IMD are unknown: sharing illicit-drugs and cannabis-cigarettes use, as well as prolonged close contact among groups of illicit-drug users (that may represents population-pockets of meningococcal carriers), is likely to increase the risk [36]. Active smoking is a well-recognized risk factor for meningococcal carriage as well as for other infections since it causes structural changes in the respiratory tract and a decrease in immune response [2, 4, 13, 17].

Meningococcal outbreaks linked to discos attendance are reported in the literature [37, 38] and it is also indicated as a risk factor for meningococcal carriage [2, 4, 13, 17]. According to these studies, in addition to the crowded conditions, several risk behaviours may converge and cumulate in these places, from smoke to illicit-drug and alcohol use, drinks and cigarettes sharing, as well as intimate kissing with multiple partners.

Higher meningococcal carriage prevalence among MSM is reported in the literature [39–41], and recently, several MenC-outbreaks involved gay, bisexual and other MSM in Europe and US [16, 42]. What promote the spread of meningococci among this population-group is partially explained by the high prevalence of risk behaviours reported in the literature among MSM that facilities frequent close-contacts, but also to an unique mechanism of sexual-transmission or a specific-susceptibility [16, 42].

No significant difference in the risk of being meningococcal carriage was found by place and month of swab collection. On this regard, the literature shows contrasting results [27–31], and a clear relation between meningococcal carriage and IMD-IR has not yet been established.

Other risk factors for meningococcal carriage reported in the literature, as alcohol-consumption, drinks-sharing, bar attendance, upper respiratory infections, antibiotics consumption, occupation and gender [2, 4, 14–17], were instead not found associated in our study or associated only at the univariate analysis.

Limits of this cross-sectional carriage survey are intrinsic to the study-design [4, 32]; in particular the convenience sampling at vaccination clinics may have introduced selection biases, as the general population might be at lower risk of carriage compared to close contacts and/or population-pockets where transmission occurs; however, the swabbing of close-contacts of MenC-cases in other studies did not find higher carriage rates [43, 44], supporting the hypothesis reported by Trotter et al. of the high recovery rate of *N. meningitidis* serogroup C strain [31]. Another limit of the study is represented by response biases that may have been introduced in sensitive questions. In addition the low number of serogroup C carriers found did not allow to identify specific risk factors and to clearly compare them with the results of the outbreak-investigation [9].

On the other hand, strengths of this study are represented by the good stability and fit of the data of multivariate models as indicated by the very similar results produced. In addition, the combined use of PCR and culture allowed maximizing the sensitivity.

## Conclusions

In conclusion, this study found a low prevalence of serogroup C carriage in an area of Tuscany affected by an outbreak due to the hypervirulent meningococcal serogroup C ST-11 (cc11) strain. Multilocus sequence typing analyses confirmed the circulation of the outbreak-clone in the outbreak-area and the risk factors analysis underlined a convergence of risk behaviours, partially confirmed by the outbreak-investigation, which should be considered in targeted immunization campaigns.

## Additional files

Additional file 1: **Table S1.** Meningococcal carriage prevalence by serogroups, demographic characteristics and risk factors. Tuscany, Italy (*n* = 110). (DOCX 42 kb)

Additional file 2: **Table S2.** Factors associated with meningococcal carriage. Univariate and multivariate analyses. Tuscany, Italy (*n* = 2285). (DOCX 41 kb)

## Abbreviations

ACWY: Quadrivalent meningococcal conjugate vaccine
AORs: Adjusted odd ratios
cc: Clonal complex
CI: Confidence intervals
IMD: Invasive meningococcal disease
IR: Incidence rate
MCC: Serogroup C conjugate vaccine
MenC: N. meningitidis serogroup C
MLST: Multilocus sequence typing
MoH: Italian Ministry of Health
MSM: Men who have sex with men
ORs: Odds ratios
PCR: Polymerase chain reaction
ST: Sequence type
STATA: Data Analysis and Statistical Software
