# Supplementary material for: Carriage rates and risk factors during an outbreak of invasive meningococcal disease due to Neisseria meningitidis serogroup C ST-11 (cc11) in Tuscany, Italy: a cross-sectional study
Source: BMC Infect Dis. 2019 Jan 8;19:29. doi: 10.1186/s12879-018-3598-3 (PMC6323866; doi:10.1186/s12879-018-3598-3)
Supplement: Supplementary file 1 — Table S1. Meningococcal carriage prevalence by serogroups, demographic characteristics and risk factors. Tuscany, Italy (n = 110). (DOCX 42 kb) [file 12879_2018_3598_MOESM1_ESM.docx]

Table 1. Meningococcal carriage prevalence by serogroups, demographic characteristics and risk factors. Tuscany, Italy (n=110^a^).

| **Variables** | **Values** | **Serogroup B**  **(n=41)** | | **Serogroup C**  **(n=4)** | | **Serogroup Y**  **(n=11)** | | **Nonencapsulated**  **(n=52)** | | **Total**  **study population**  **(n=2,285)** |
| --- | --- | --- | --- | --- | --- | --- | --- | --- | --- | --- |
|  |  | **N** | **Prevalence %**  **(95%CI)** | **N** | **Prevalence %**  **(95%CI)** | **N** | **Prevalence %**  **(95%CI)** | **N** | **Prevalence %**  **(95%CI)** | **N** |
| Gender | Male | 17 | 1.8  (1.1-2.9) | 2 | 0.2  (0.0-0.9) | 7 | 0.7  (0.3-1.5) | 20 | 2.1  (1.8-3.0) | 940 |
|  | Female | 24 | 1.8  (1.1-2.6) | 2 | 0.1  (0.0-0.4) | 4 | 0.3  (0.1-0.8) | 32 | 2.4  (2.0-3.1) | 1,345 |
| Age group | 11-19 | 23 | 2.8  (1.8-4.1) | 4 | 0.5  (0.1-1.2) | 7 | 0.8  (0.3-1.7) | 13 | 1.6  (1.1-1.9) | 828 |
|  | 20-30 | 13 | 3.0  (1.6-5.1) | 0 | 0.0  (0.0-1.4) | 2 | 0.5  (0.1-1.7) | 24 | 5.5  (3.1-6.4) | 434 |
|  | 31-45 | 5 | 0.5  (0.2-1.1) | 0 | 0.0  (0.0-0.4) | 2 | 0.2  (0.0-0.7) | 15 | 1.5  (0.9-2.2) | 1,023 |
| Place swab  collection | Siena | 11 | 2.2  (1.1-3.9) | 0 | 0.0  (0.0-0.7) | 4 | 0.8  (0.2-2.0) | 13 | 2.6  (1.9-4.1) | 497 |
|  | Grosseto | 8 | 1.3  (0.6-2.6) | 1 | 0.2  (0.0-4.0) | 3 | 0.5  (0.1-1.5) | 13 | 2.2  (1.5-3.7) | 597 |
|  | Firenze | 10 | 1.7  (0.8-3.1) | 0 | 0.0  (0.0-0.6) | 0 | 0.0  (0.0-0.6) | 11 | 1.9  (1.0-4.1) | 580 |
|  | Empoli | 12 | 2.0  (0.8-3.0) | 3 | 0.5  (0.1-1.4) | 4 | 0.7  (0.2-1.7) | 15 | 2.5  (1.7-3.1) | 611 |
| Month swab  collection | March | 5 | 1.6  (0.5-3.7) | 2 | 0.6  (0.1-2.3) | 0 | 0.0  (0.0-1.2) | 13 | 4.1  (3.3-5.6) | 314 |
|  | April | 18 | 1.7  (1.0-2.7) | 0 | 0.0  (0.0-0.3) | 4 | 0.4  (0.1-1.0) | 11 | 1.0  (0.1-2.6) | 1,053 |
|  | May | 18 | 2.3  (1.3-3.6) | 2 | 0.3  (0.0-0.9) | 7 | 0.9  (0.4-1.8) | 14 | 1.8  (0.6-2.4) | 796 |
|  | June | 0 | 0.0  (0.0-22.3) | 0 | 0.0  (0.0-3.0) | 0 | 0.0  (0.0-3.0) | 14 | 11.5  (9.4-15.3) | 122 |
| Occupation | Teacher | 1 | 2.9  (0.1-14.9) | 0 | 0.0  (0.0-10.0) | 0 | 0.0  (0.0-10.0) | 9 | 25.7  (14.4-35.1) | 35 |
|  | Student | 31 | 2.5  (1.7-3.5) | 4 | 0.3  (0.1-0.8) | 8 | 0.6  (0.3-1.2) | 9 | 0.7  (0.1-1.2) | 1,263 |
|  | Restaurant,  bar, pub | 0 | 0.0  (0.0-6.4) | 0 | 0.0  (0.0-6.4) | 0 | 0.0  (0.0-6.4) | 12 | 21.4  (13.2-33.7) | 56 |
|  | Armed  forces | 0 | 0.0  (0.0-9.5) | 0 | 0.0  (0.0-12.8) | 0 | 0.0  (0.0-12.8) | 8 | 29.6  (17.4-40.1) | 27 |
|  | Health  professional | 1 | 0.7  (0.0-4.1) | 0 | 0.0  (0.0-2.7) | 0 | 0.0  (0.0-2.7) | 9 | 6.7  (2.0-10.3) | 135 |
|  | Other | 8 | 1.0  (0.5-2.0) | 0 | 0.0  (0.0-0.59 | 3 | 0.4  (0.1-1.1) | 5 | 0.7  (0.1-4.8) | 769 |
| Drinking  risk level | Low | 36 | 1.8  (1.3-2.5) | 3 | 0.1  (0.0-0.4) | 11 | 0.5  (0.3-1.0) | 26 | 1.3  (0.4-8.5) | 2,012 |
|  | Moderate | 5 | 1.9  (0.6-4.3) | 1 | 0.4  (0.0-2.1) | 0 | 0.0  (0.0-1.4) | 26 | 9.7  (6.1- 13.5) | 269 |
|  | High | 0 | 0.0  (0.0-60.2) | 0 | 0.0  (0.0-60.2) | 0 | 0.0  (0.0-60.2) | 0 | 0.0  (0.00-60.2) | 4 |
| Sharing  drinks^b^ | No | 17 | 1.2  (0.7-1.9) | 0 | 0.0  (0.0-0.3) | 7 | 0.5  (0.2-1.0) | 35 | 2.5  (1.3-6.9) | 1,395 |
|  | Yes | 24 | 2.7  (1.7-4.0) | 4 | 0.4  (0.1-1.1) | 4 | 0.4  (0.1-1.1) | 17 | 1.9  (0.4-3.1) | 890 |
| Illicit drugs  consumption^b^ | No | 38 | 1.7  (1.2-2.3) | 3 | 0.1  (0.0-5.2) | 7 | 0.3  (0.1-0.6) | 49 | 2.1  (0.8-3.7) | 2,250 |
|  | Yes | 3 | 8.6  (1.8-22.5) | 1 | 2.9  (0.5-14.2) | 4 | 11.4  (3.1-26.1) | 3 | 8.6  (4.7-87.6) | 35 |
| Active  smoking | No | 25 | 1.4  (0.9-2.0) | 3 | 0.2  (0.0-0.12) | 5 | 0.3  (0.1-0.7) | 20 | 1.1  (0.1-8.3) | 1,841 |
|  | Yes | 16 | 3.6  (2.1-5.8) | 1 | 0.2  (0.2-2.3) | 6 | 1.4  (0.4-2.6) | 32 | 7.2  (2.6-17.9) | 444 |
| Passive  smoking | No | 22 | 1.5  (0.9-2.3) | 0 | 0.0  (0.0-0.3) | 6 | 0.4  (0.2-0.9) | 40 | 2.7  (2.0-3.1) | 1,465 |
|  | Yes | 19 | 2.3  (1.4-3.6) | 4 | 0.5  (0.1-1.2) | 5 | 0.6  (0.2-1.4) | 12 | 1.5  (1.0-2.6) | 820 |
| Sexual  intercourses^b^ | No | 3 | 0.9  (0.3-3.1) | 4 | 1.2  (0.0-3.1) | 3 | 0.9  (0.2-2.6) | 12 | 3.6  (2.2-6.3) | 335 |
|  | Same-sex | 1 | 4.8  (0.1-20.4) | 0 | 0.0  (0.0-0.6) | 1 | 4.8  (0.1-20.4) | 16 | 76.2  (34.2-94.0) | 21 |
|  | Heterosexual | 37 | 1.9  (1.3-2.6) | 0 | 0.0  (0.0-0.2) | 7 | 0.4  (0.1-0.7) | 24 | 1.2  (0.4-1.9) | 1,929 |
| Attending  bar, restaurants, pub^b^ | No | 17 | 1.9  (1.1-3.0) | 2 | 0.2  (0.0-0.8) | 4 | 0.4  (0.1-1.1) | 22 | 2.5  (1.9-3.0) | 892 |
|  | Yes | 24 | 1.7  (1.1-2.6) | 2 | 0.1  (0.0-0.5) | 7 | 0.5  (0.2-1.0) | 30 | 2.2  (1.8-2.8) | 1,393 |
| Attending disco,  clubs, parties^b^ | No | 7 | 0.8  (0.3-1.6) | 0 | 0.0  (0.0-0.4) | 1 | 0.1  (0.0-0.6) | 12 | 1.4  (1.0-2.0) | 886 |
|  | Yes | 34 | 2.4  (1.7-3.4) | 4 | 0.3  (0.1-0.7) | 10 | 0.7  (0.3-1.3) | 40 | 2.9  (2.3-4.1) | 1,399 |
| Attending other  close groups^b^ | No | 12 | 1.1  (0.6-2.0) | 2 | 0.2  (0.0-0.7) | 5 | 0.5  (0.2-1.1) | 27 | 2.5  (2.0-3.1) | 1,068 |
|  | Yes | 29 | 2.4  (1.6-3.4) | 2 | 0.2  (0.0-0.6) | 6 | 0.5  (0.2-1.1) | 25 | 2.1  (1.8-3.3) | 1,217 |
| Upper respiratory  tract infections^b^ | No | 32 | 1.7  (1.1-2.3) | 2 | 0.1  (0.0-0.4) | 9 | 0.5  (0.2-0.9) | 33 | 1.7  (1.1-2.3) | 1,920 |
|  | Yes | 9 | 2.5  (1.1-4.6) | 2 | 0.5  (0.1-2.0) | 2 | 0.5  (0.1-2.0) | 19 | 5.2  (4.2-7.1) | 365 |
| Antibiotic  consumption^b^ | No | 37 | 1.8  (1.3-2.5) | 4 | 0.2  (0.1-0.5) | 11 | 0.5  (0.3-1.0) | 36 | 1.8  (1.3-2.5) | 2,031 |
|  | Yes | 4 | 1.6  (0.4-4.0) | 0 | 0.0  (0.0-1.4) | 0 | 0.0  (0.0-1.4) | 16 | 6.3  (3.6-9.0) | 254 |
| ^a^ One carrier of *N. meningitidis* serogroup E and one of serogroup Z not shown  ^b^ In the month before swab collection | | | | | | | | | | |
