# Supplementary material for: Carriage rates and risk factors during an outbreak of invasive meningococcal disease due to Neisseria meningitidis serogroup C ST-11 (cc11) in Tuscany, Italy: a cross-sectional study
Source: BMC Infect Dis. 2019 Jan 8;19:29. doi: 10.1186/s12879-018-3598-3 (PMC6323866; doi:10.1186/s12879-018-3598-3)
Supplement: Supplementary file 2 — Table S2. Factors associated with meningococcal carriage. Univariate and multivariate analyses. Tuscany, Italy (n = 2285). (DOCX 41 kb) [file 12879_2018_3598_MOESM2_ESM.docx]

Table 2. Factors associated with meningococcal carriage. Univariate and multivariate analyses. Tuscany, Italy (n=2,285).

| Variables | Values | Positive  (n=110) | Negative  (n= 2,157) | Total  (n=2,285) | Univariate  analysis | | Multivariate  analysis^a^ | | Backward  model^b^ | |
| --- | --- | --- | --- | --- | --- | --- | --- | --- | --- | --- |
|  |  | N  (%) | N  (%) | N | OR  (95% CI) | p-value | AOR  (95% CI) | p-value | AOR  (95% CI) | p-value |
| Gender | Male | 47  (42.7) | 893  (41.1) | 940 | REF | 0.31 | REF | 0.76 | REF | 0.58 |
|  | Female | 63  (57.3) | 1,282  (58.9) | 1,345 | 0.77  (0.46-1.29) |  | 1.09  (0.62-1.90) |  | 1.00  (0.46-7.50) |  |
| Age group | 31-45 | 20  (18.2) | 808  (37.1) | 828 | REF | <0.01 | REF | 0.01 | REF | 0.02 |
|  | 20-30 | 40  (36.4) | 394  (18.1) | 434 | 4.49  (1.83-11.00) |  | 3.08  (1.97-9.79) |  | 3.12  (1.23-7.91) |  |
|  | 11-19 | 50  (45.5) | 973  (44.7) | 1,023 | 4.28  (1.89-9.66) |  | 3.86  (0.98-15.1) |  | 2.12  (0.96-17.51) |  |
| Place swab collection | Siena | 28  (25.5) | 469  (21.6) | 497 | REF | 0.26 | NI | NI | NI | NI |
|  | Grosseto | 26  (23.6) | 571  (26.3) | 597 | 0.72  (0.34-1.52) |  |  |  |  |  |
|  | Firenze | 22  (20.0) | 558  (25.7) | 580 | 0.62  (0.28-1.37) |  |  |  |  |  |
|  | Empoli | 34  (30.9) | 577  (26.5) | 611 | 1.09  (0.55-2.15) |  |  |  |  |  |
| Month swab collection | March | 20  (18.2) | 294  (13.5) | 314 | REF | 0.84 | NI | NI | NI | NI |
|  | April | 35  (31.8) | 1,018  (46.8) | 1,053 | 0.92  (0.44-2.40) |  |  |  |  |  |
|  | May | 41  (37.3) | 755  (34.7) | 796 | 1.60  (0.69-3.70) |  |  |  |  |  |
|  | June | 14  (12.7) | 108  (5.0) | 122 | 1.26  (0.00-3.78) |  |  |  |  |  |
| Occupation | Teachers | 10  (9.1) | 25  (1.1) | 35 | REF | 0.33 | NI | NI | NI | NI |
|  | Students | 54  (49.1) | 1,209  (55.6) | 1,263 | 0.60  (0.34-5.06) |  |  |  |  |  |
|  | Restaurant, bar, pub | 12  (10.9) | 44  (2.0) | 56 | 1.20  (0.00-5.13) |  |  |  |  |  |
|  | Armed  forces | 8  (7.3) | 19  (0.9) | 27 | 1.41  (0.01-10.67) |  |  |  |  |  |
|  | Health professional | 10  (9.1) | 125  (5.7) | 135 | 1.51  (0.07-4.02) |  |  |  |  |  |
|  | Other | 16  (14.5) | 753  (34.6) | 769 | 1.02  (0.43-9.12) |  |  |  |  |  |
| Drinking risk level | Low | 78  (70.9) | 1,934  (88.9) | 2,012 | REF | 0.67^d^ | NI | NI | NI | NI |
|  | Moderate | 32  (29.1) | 237  (10.9) | 269 | 1.84  (0.36-1.98) |  |  |  |  |  |
|  | High | 0  (0.0) | 4  (0.2) | 4 | 0.73  (0.00-57.0) |  |  |  |  |  |
| Sharing drinks^c^ | No | 61  (55.5) | 1,334  (61.3) | 1,395 | REF | <0.01 | REF | 0.68 | NI | NI |
|  | Yes | 49  (44.5) | 841  (38.7) | 890 | 2.03  (1.20-3.41) |  | 1.13  (0.63-2.06) |  |  |  |
| Illicit drugs consumption^c^ | No | 96  (87.3) | 2,154  (99.0) | 2,250 | REF | <0.01 | REF | <0.01 | REF | <0.01 |
|  | Yes | 14  (12.7) | 21  (1.0) | 35 | 20.18  (9.39-43.34) |  | 6.30  (2.44-16.32) |  | 6.07  (2.39-15.42) |  |
| Active smoking | No | 52  (47.3) | 1,789  (82.3) | 1,841 | REF | <0.01 | REF | 0.01 | REF | <0.01 |
|  | Yes | 58  (52.7) | 386  (17.7) | 444 | 3.41  (2.02-5.76) |  | 2.78  (1.37-5.63) |  | 2.79  (1.42-5.45) |  |
| Passive smoking | No | 70  (63.6) | 1,395  (64.1) | 1,465 | REF | 0.25 | NI | NI | NI | NI |
|  | Yes | 40  (36.4) | 780  (35.9) | 820 | 1.64  (0.77-2.75) |  |  |  |  |  |
| Sexual intercourses^c^ | No | 22  (20.0) | 313  (14.4) | 335 | REF | <0.01 | REF | <0.01 | REF | <0.01 |
|  | Same-sex | 18  (16.4) | 3  (0.1) | 21 | 13.15  (5.05-34.26) |  | 6.69  (2.16-20.70) |  | 6.90  (2.29-20.84) |  |
|  | Heterosexual | 70  (63.6) | 1,859  (85.5) | 1,929 | 0.73  (0.36-1.47) |  | 0.85  (0.15-19.13) |  | 0.79  (0.23-15.36) |  |
| Attending bar, restaurants, pub^c^ | No | 45  (40.9) | 847  (38.9) | 892 | REF | 0.95 | NI | NI | NI | NI |
|  | Yes | 65  (59.1) | 1,328  (61.1) | 1,393 | 1.00  (0.59-1.70) |  |  |  |  |  |
| Attending disco, clubs, parties^c^ | No | 23  (20.9) | 863  (39.7) | 886 | REF | <0.01 | REF | 0.04 | REF | 0.03 |
|  | Yes | 87  (79.1) | 1,312  (60.3) | 1,399 | 2.83  (1.46-5.47) |  | 2.06  (1.04-4.23) |  | 2.13  (1.06-4.30) |  |
| Attending other close groups^c^ | No | 47  (42.7) | 1,021  (46.9) | 1,068 | REF | 0.84 | NI | NI | NI | NI |
|  | Yes | 63  (57.3) | 1,154  (53.1) | 1,217 | 1.73  (0.91-2.99) |  |  |  |  |  |
| Upper respiratory tract infections^c^ | No | 77  (70.0) | 1,843  (84.7) | 1,920 | REF | 0.72 | NI | NI | NI | NI |
|  | Yes | 33  (30.0) | 332  (15.3) | 365 | 1.13  (0.57-2.27) |  |  |  |  |  |
| Antibiotic consumption^c^ | No | 90  (81.8) | 1,941  (89.2) | 2,031 | REF | 0.26 | NI | NI | NI | NI |
|  | Yes | 20  (18.2) | 234  (10.8) | 254 | 1.06  (0.20-2.55) |  |  |  |  |  |
| OR: odds ratio; AOR: adjusted odds ratio; CI: confidence interval; REF: reference value; NI: not included  ^a^Multivariate regression model including variables with p<0.20 at the univariate analysis. Age and sex included independently from the p-value  ^b^Stepwise backward elimination at p>0.10 threshold. Age and sex included independently from the p-value  ^c^In the month before swab collection  ^d^Exact logistic regression | | | | | | | | | | |
